# Mental health trajectories over the COVID-19 pandemic among young adults reporting adverse childhood experiences

**DOI:** 10.3389/fpubh.2025.1546409

**Published:** 2025-07-09

**Authors:** Behnam Mousavi, Jessy Moore, Karen A. Patte, William Pickett, Deborah D. O’Leary, Terrance J. Wade

**Affiliations:** Department of Health Sciences, Brock University, St Catharines, ON, Canada

**Keywords:** adverse childhood experiences, ACEs, young adults, mental health, COVID-19, stress

## Abstract

**Background:**

Higher exposure to adverse childhood experiences (ACEs) has been shown to worsen the effect of COVID-19 stress on mental health problems in the early phase of the COVID-19 pandemic among young adults. This study extends that research by examining depression, anxiety, hostility, and perceived stress trajectories across successive phases of the COVID-19 pandemic in a prospective, multi-wave panel study using data collected pre-COVID-19 pandemic onset, Early pandemic, Peak pandemic, and Post-Peak pandemic.

**Methods:**

The baseline data come from the Niagara Longitudinal Heart Study (NLHS) and the three COVID-19 waves come from a sub-study of the NLHS examining the specific impact of the pandemic. Using a Bayesian multivariate mixed-model regression framework, 171 participants who responded to at least one wave of the COVID-19 sub-study were included.

**Results:**

Participants with higher ACE scores and high COVID-19 stress had elevated trajectories of several poor mental health measures that stayed higher than other groups across all waves of data collection.

**Discussion:**

Young adults who reported higher ACEs were more susceptible to subsequent stress exposure, highlighting a specific, high-risk group who may benefit from targeted intervention programs during times of crisis such as the COVID-19 pandemic.

## 1 Introduction

Adverse Childhood Experiences (ACEs), encompassing maltreatment and severely dysfunctional living situations in childhood, have been identified to have a lasting impact on physical and mental health across the life course (1–3). ACEs commonly include experiences such as physical, emotional, or sexual abuse; physical or emotional neglect; and household challenges like parental substance use, mental illness, incarceration, domestic violence, or separation/divorce. These early life adversities disrupt normal development and can impair emotional regulation, stress response systems, and attachment patterns, thereby increasing vulnerability to mental health disorders later in life (4, 5). Importantly, research findings consistently emphasize the cumulative effect of ACEs, with individuals exposed to more types of ACEs at a heightened risk of developing mental health disorders such as depression, anxiety, and post-traumatic stress disorder (PTSD); physical health issues including cardiovascular disease and obesity; and detrimental lifestyles, such as substance abuse and engagement in risky behaviors (6). Moreover, the prevalence of ACEs is more widespread than commonly believed, with numerous studies across Canada, the United States, and Europe, indicating that a significant portion of the population has experienced multiple ACEs (7–10). The enduring effect on both physical and mental health has been well documented in the literature, with ACEs constituting a significant public health concern (6).

The COVID-19 pandemic, declared in March 2020, and the resulting government interventions—such as lockdowns, school closures, and isolation mandates—constituted a population-level crisis with broad health and social impacts. As of 2024, the World Health Organization has reported more than 770 million confirmed cases and nearly 7 million deaths globally (11). The emergence of variant strains such as Alpha, Beta, Gamma, Delta, and Omicron added ongoing uncertainty regarding transmissibility and vaccine protection (12, 13). The various governmental responses to control the spread of COVID-19 included lockdowns, closures, and forced isolation, which led to widespread concern about potential mental health consequences. Some studies have indicated that the pandemic contributed to elevated rates of mental health issues such as anxiety, depression, and PTSD both among individuals with early COVID-19 infections (14, 15) as well as many who did not contract the virus or did so later on in the pandemic (16). Cross-sectional and longitudinal research among adolescents and adults has found that those with higher ACEs were more vulnerable to the negative consequences of the pandemic and related governmental responses. This resulted in higher levels of anxiety and depression early in the pandemic across studies of various groups including adolescents (17, 18) adults (19–22), and young adults (23). This evidence suggests that ACEs may increase the negative impact of pandemic-related stress.

Young adults, as a cohort, are in an especially precarious position compared to older adults due to their life stage and general higher prevalence of psychological problems (24). Pandemic-related stressors—such as social isolation from post-secondary school closures, transitions to online learning, economic hardship due to job losses in service industries, and widespread misinformation—disproportionately impacted this age group (25). A prospective study that examined the interaction between ACEs and COVID-19 stressors on mental health among young adults early in the pandemic found that those with high ACEs and high COVID-19 stress had greater increase in mental health problems compared to those reporting lower pandemic stress and/or lower ACEs (23).

While informative, existing studies are mainly focused on only the early phase of the COVID-19 pandemic. Further research is needed to examine changes in mental health over the full duration of the pandemic as both the infection rates and government responses evolved. By utilizing a prospective, longitudinal approach with pre-pandemic, baseline data, this study extends previous work that focused on the early pandemic phase. The current study examines the relationship between ACEs and COVID-19 stressors on changes in mental health measures across multiple phases of data collection occurring in the Early, Peak, and Post-Peak phases of the pandemic. Examining these relationships across multiple phases of data collection allows us to assess the longer-term mental health trajectories among young adults. These results may be valuable to assess more generally whether people with high exposure to ACEs may exacerbate the negative mental health effects of persistent stressful experiences across the life course.

## 2 Methods

### 2.1 Study sample

This prospective panel study included 4 waves of data collection. The pre-COVID-19 baseline data comes from the Niagara Longitudinal Heart Study (NLHS) (26), which recruited 248 participants aged 18 years or older from the Niagara region prior to the university-wide COVID-19 shutdown of human research at Brock University on March 17, 2020. In addition to the comprehensive testing protocol that included anthropometric, non-invasive cardiovascular, and biological specimen data collection, the NLHS incorporated a detailed self-reported questionnaire that collected information on ACEs and various mental health domains.

### 2.2 Study procedures

The NLHS-COVID-19 sub-study included three waves of data collected using an online Qualtrics XM survey that included all mental health measures collected at baseline as well as measures on various COVID-19 stressors. These waves will be referred to as the Early, Peak, and Post-Peak phases. The first COVID-19 wave of data (Early phase) was collected between July and October, 2020. All 248 participants in the NLHS consented to be contacted for inclusion in future research and were contacted via email and social media platforms and invited to take part in the online survey. A total of 171 participants completed the Early phase survey, resulting in a response rate of 69%. The second COVID-19 wave survey (Peak phase) was administered between January and March, 2021 during the period of one of the highest infection rates, strict government restrictions, and limited, priority access to vaccines. 161 participants completed surveys during this phase for a response rate of 94% from the Early phase (65% from baseline). The Post-Peak phase of data was collected between March and April, 2022 when the majority of the population had received vaccinations, infection rates were low and declining, and most government lockdown and masking requirements had been relaxed or removed. 138 participants completed surveys during the Post-Peak phase, resulting in a response rate of 80% from the Early phase (56% from baseline).

### 2.3 Ethical considerations

Participants provided active written consent to participate in the NLHS study and were compensated $100 for their involvement. All participants also provided written consent to be re-contacted for future studies. Active consent to participate in the COVID-19 sub-study was secured through email and by clicking on the link taking them to the online survey. Participants were compensated $20 for each wave of the COVID-19 sub-study completed. To ensure confidentiality, all data were anonymized prior to analysis. Personal identifying information, such as names and contact details, were stored separately from survey responses and accessible only to designated research staff. Participant data were coded using unique identification numbers, and any published results were reported in aggregate form to prevent individual identification. Both the NLHS and the COVID-19 sub-study received Brock University—Health Research Ethics Board (HREB) approvals (#18–288; #20–313).

### 2.4 Study measures

Adverse childhood experiences were assessed retrospectively in the NLHS survey using the CTES 2.0 questionnaire, designed for children and adolescents (27, 28). Although originally developed for younger populations, the CTES 2.0 has been used in retrospective self-report by young adults and has demonstrated acceptable internal consistency and construct validity in this context (29). It captures a broad spectrum of ACEs, including various forms of maltreatment and severe household dysfunction. To ensure alignment with the widely used Kaiser ACEs questionnaire (2), we included 14 CTES items that map onto eight ACE domains: emotional (2 items), physical (1 item), and sexual abuse (2 items), witnessing intimate partner violence (2 items), family member mental illness or suicidal ideation (2 items), substance abuse (2 items), incarceration (1 item), and unexpected parental separation (2 items). A domain was scored as positive if any associated item was endorsed.

The cumulative ACE score ranged from 0 to 4+, consistent with prior research (2). This 4 + ACEs threshold is widely used in both early and recent studies [e.g., (9, 30)] to identify individuals at elevated risk of poor outcomes. In our own data, we observed that mental health scores plateaued beyond 4 ACEs, suggesting no additional effect beyond this point and supporting the use of the 4 + cutoff.

The CTES 2.0 in our sample demonstrated acceptable internal consistency, with Cronbach’s alpha ranging from 0.70 to 0.84 across domains, consistent with previous studies (29).

COVID-19 stressors were grouped into 5 domains—Emotional, Family and Financial, Lifestyle, Substance Use, and Community Support—based on an exploratory factor analysis (EFA) of the first wave of COVID-19 data (see Supplementary Table S1). Emotional stressors included five items (e.g., loneliness, frustration, suspicion); Lifestyle stressors (2 items) focused on inactivity and diet changes; Substance Use (2 items) captured increased alcohol/drug use; Family and Financial (5 items) covered conflict, income loss, and instability; and Community Support (3 items) reflected access difficulties. These groupings were derived via EFA and not from previously validated subscales. We calculated weighted mean scores for each domain using item-specific factor loadings to account for differential contribution to the latent stress construct.

The overall internal consistency across stressor domains in our sample was high (Cronbach’s alpha = 0.84), and the EFA structure showed good factor loadings (see Supplementary Table S1).

Mental health outcomes were assessed at baseline (pre-COVID-19) and in each COVID-19 wave. Depression was measured using the 20-item Center for Epidemiological Studies–Depression Scale [CES-D; (31)]. The CES-D has demonstrated strong reliability (Cronbach’s alpha = 0.87–0.89 in recent studies (30) and construct validity across adult samples (32). Anxiety and hostility were measured using subscales of the Symptom Checklist-90-R (SCL-90R (33);). These subscales have shown high internal consistency (α > 0.85) and strong validity in both clinical and non-clinical populations (34). Perceived stress was measured using the 14-item Perceived Stress Scale (PSS (35);), with recent studies reporting high internal consistency (α = 0.86–0.89) and convergent validity with anxiety and depression (36, 37).

Age, sex, and education were included as covariates in all regression models. These were collected at baseline.

### 2.5 Statistical analysis

The analysis proceeded in four steps including attrition analysis, descriptive analysis, correlation analysis over time, and Bayesian multivariate mixed-model regression analyses that adjusted for missing data over waves as well as skewed standard errors and intraclass correlations (ICC) within participants. Attrition analyses employed a fixed-effect, longitudinal regression approach that included indicators for participants who remained in the study and those who dropped out at each data time point. Regression analyses included interactions between ACEs and each COVID-19 stressor examined over time to gauge their conditional effects on changes in mental health outcomes. Since the NLHS pre-pandemic baseline did not include COVID-19 stressors, we used the change in mental health measures from the baseline to the Early phase as the reference category. We then examined two sets of three-way interaction variables (ACEs × COVID-19 stressor × Peak; ACEs × COVID-19 stressor × Post-Peak) to compare the subsequent changes between the Early and Peak phases, as well as between the Peak and Post-Peak phases.

In our statistical power analysis of the Bayesian multivariate mixed-effects model, we utilized the methodology outlined by Johnson et al. (38) and Thomas and Juanes (39) to estimate a power of 0.71 for detecting the interaction of Covid stressor, ACE, and time. This power suggests a relatively strong ability to detect true effects, indicating a good potential to avoid false negatives in the population. All analyses were conducted using the statistical software R [v 4.3.1; (40)].

## 3 Results

The attrition analysis included all mental health outcomes and covariates and is detailed in Supplementary Table S2. All variables except for education were non-significant. Those who discontinued participation at any point across COVID-19 sub-study data waves had lower average education compared to those who remained in the study (*p* < 0.05). Slight differences in mean ACE scores across waves are due to participant attrition rather than true change in exposure. ACEs were only measured at baseline; changes in sample composition over time explain the small shifts observed.

Descriptive statistics analysis identified significant differences over time in depression, hostility, and anxiety but not for perceived stress. This is consistent when using all valid cases for each wave and when using the final sample of 138. The changes indicate depression, anxiety, and hostility scores increased from baseline to the Early phase, peaked during the Peak phase, and declined again in the Post-Peak phase. In contrast, perceived stress remained stable across all phases. Average COVID-19 stressor scores increased from Early to Peak and declined in the Post-Peak wave, consistent with the pattern observed in mental health outcomes (Table 1).

**Table 1:** Descriptive statistics of demographic characteristics, mental health outcomes, ACEs and COVID-19 stressors at pre-COVID-19 (baseline), Early, Peak, and Post-Peak phases of the pandemic in a prospective study of young adults.

	Pre-COVID-19	Early	Peak	Post-Peak	*p*-value
Participants in each study wave					
*N*	248	171	161	138	
Sex, %					
Male	42.69	-	-	-	
Female	57.31	-	-	-	
Age, years, mean (SD)	22.11 (1.49)	-	-	-	
Education, %					
Grade 12 or less	8.77	-	-	-	
High School diploma (or GED)	19.88	-	-	-	
Partial college/training	21.05	-	-	-	
College/university degree	43.86	-	-	-	
Graduate/professional degree	6.43	-	-	-	
ACEs score, mean (SD)	2.08 (1.46)	1.98 (1.39)	1.92 (1.39)	1.96 (1.39)	
ACEs score, *n* (%)					
0	43 (17.33)	28 (16.37)	28 (17.39)	24 (17.39)	
1	60 (24.20)	44 (25.73)	43 (26.71)	34 (24.64)	
2	48 (19.35)	40 (23.39)	37 (22.98)	32 (23.19)	
3	30 (12.10)	21 (12.28)	19 (11.80)	19 (13.77)	
4 or more	67 (27.02)	38 (22.22)	34 (21.12)	29 (21.01)	
COVID-19 Stressors, mean (SD)					
Emotional	-	8.81 (2.64)	10.70 (2.77)	8.73 (2.82)	0.000
Family and financial	-	11.06 (1.94)	11.75 (1.89)	11.39 (1.69)	0.000
Community support	-	6.27 (1.44)	6.49 (1.45)	6.44 (1.27)	0.023
Lifestyle	-	4.73 (1.80)	5.03 (1.71)	4.49 (1.72)	0.000
Substance use	-	4.79 (1.01)	5.07 (0.95)	4.87 (1.03)	0.000
Outcome Measures, mean (SD)					
Depression	15.76 (10.93)	17.17 (12.23)	18.24 (12.94)	15.4 (10.86)	0.004
Anxiety	16.43 (5.39)	17.81 (6.01)	16.99 (6.15)	16.39 (5.45)	0.003
Hostility	9.31 (2.61)	10.08 (3.01)	9.64 (2.89)	9.19 (2.51)	0.000
Perceived Stress	26.77 (5.82)	27.41 (6.50)	27.26 (6.90)	26.75 (6.85)	0.670
Participants with data across all study waves					
*N*	138	138	138	138	
Sex, %					
Male	41.47	-	-	-	
Female	58.53	-	-	-	
Age, years, mean (SD)	20.12 (0.94)	-	-	-	
Education, %					
Grade 12 or less	7.28	-	-	-	
High School diploma (or GED)	17.1	-	-	-	
Partial college/training	20.32	-	-	-	
College/university degree	49.60	-	-	-	
Graduate/professional degree	5.7	-	-	-	
ACEs score, mean (SD)	1.96 (1.39)	-	-	-	
ACEs score, *n* (%)					
0	24 (17.4)	-	-	-	
1	34 (24.6)	-	-	-	
2	32 (23.2)	-	-	-	
3	19 (13.8)	-	-	-	
4 or more	29 (21.0)	-	-	-	
COVID-19 Stressors, mean (SD)					
Emotional	-	8.83 (2.64)	10.69 (2.77)	8.73 (2.82)	0.000
Family and financial	-	11.08 (1.92)	11.77 (1.81)	11.39 (1.69)	0.000
Community support	-	6.28 (1.47)	6.52 (1.43)	6.44 (1.27)	0.101
Lifestyle	-	4.76 (1.77)	5.07 (1.71)	4.49 (1.72)	0.000
Substance use	-	4.80 (0.98)	5.03 (0.96)	4.87 (1.03)	0.004
Outcome Measures, mean (SD)					
Depression	15.24 (11.19)	16.46 (12.45)	18.18 (13.17)	15.4 (10.86)	0.005
Anxiety	16.82 (5.2)	17.07 (6.48)	17.82 (6.28)	16.39 (5.45)	0.036
Hostility	9.20 (2.43)	9.60 (3.06)	9.93 (2.99)	9.19 (2.51)	0.005
Perceived Stress	26.73 (5.99)	26.86 (6.45)	27.13 (6.98)	26.75 (6.85)	0.855

The p-values in this table reflect the results of repeated measures ANOVAs across the three COVID-19 study phases (early, peak, post-peak). Demographic variables (sex, age, education) and baseline ACE scores were only collected at the pre-COVID-19 phase and therefore do not have repeated measures p-values. The mean ACE score is reported at each time point, based on the number of participants who responded to the COVID-19 survey and who were therefore included in the analysis. The “Participants with data across all study waves” section includes only those who completed all four waves of the study. SD = standard deviation.

Before applying a Bayesian multivariate mixed-effects model, it was important to first examine the correlations among the response variables—changes in depression, anxiety, hostility, and perceived stress—since the model assumes these outcomes are interrelated. The correlation analysis across the three waves, presented in Table 2, revealed a consistent and strong association between changes in depression and perceived stress: r = 0.531 (pre- to Early), r = 0.496 (Early to Peak), and r = 0.563 (Peak to Post-Peak). These correlations represent co-variation in symptom changes between adjacent time points.

**Table 2:** Correlation analysis of mental health changes across the pandemic.

Wave	`	Depression	Hostility	Anxiety	Perceived Stress	*p*-Value
Pre-Pandemic to Early Pandemic	Depression	1				<0.05
	Hostility	0.318	1			<0.05
	Anxiety	0.375	0.412	1		<0.05
	Perceived Stress	0.531	0.302	0.400	1	<0.05
Early Pandemic to Peak Pandemic	Depression	1				<0.05
	Hostility	0.237	1			<0.05
	Anxiety	0.457	0.501	1		<0.05
	Perceived Stress	0.496	0.207	0.336	1	<0.05
Peak Pandemic to Post Peak Pandemic	Depression	1				<0.05
	Hostility	0.309	1			<0.05
	Anxiety	0.552	0.524	1		<0.05
	Perceived Stress	0.563	0.192	0.330	1	<0.05

This table presents Pearson correlation coefficients among depression, anxiety, hostility, and perceived stress across different phases of the COVID-19 pandemic. The p-values indicate the significance of these correlations within each phase. These associations are consistent with established literature and reflect the interrelated nature of these mental health variables.

Anxiety also emerged as a central construct in this network of symptoms. Correlations between anxiety and depression increased over time: r = 0.375, 0.457, and 0.552 across the three intervals. Similarly, the relationship between anxiety and hostility strengthened across phases: r = 0.412, 0.501, and 0.524, peaking during the Early-to-Peak pandemic period. These results indicate that as anxiety levels changed during the pandemic, they became increasingly synchronized with changes in both depression and hostility. Finally, although the correlation between hostility and perceived stress was weaker overall (r = 0.302, 0.207, 0.192), it remained statistically significant in each wave, suggesting a modest but consistent association.

In the final phase of the analysis, a Bayesian multivariate mixed-effects model was used to investigate how COVID-19 stressors and ACEs interacted to affect mental health changes over time. A summary of the regression results is provided in Table 3, with the full regression details available in Supplementary Table S3. Although the main effect of family and financial stress appeared negative (β = −6.18), this should be interpreted alongside the significant interaction with ACEs (β = 0.73), which indicates that the impact of stress varies by ACE exposure. Among individuals with higher ACEs, greater family and financial stress was associated with increased depression symptoms. Additionally, the three-way interaction with time (β = −0.99) suggests that while the influence of family and financial stress on depression weakens over time, it remains more protective during the Peak to Post-Peak pandemic period compared to the pre-pandemic period. This finding aligns with the correlation results, where depression consistently correlates strongly with perceived stress, suggesting that perceived stress may play a mediating role in the relationship between family and financial stress and depression.

**Table 3:** Summary of significant results from Bayesian multivariate mixed-effects regression predicting changes in mental health outcomes based on the interaction between COVID-19 stressors and aces across Early, Peak, and Post-Peak pandemic phases.

Response	Covariates	Beta	SE	l-95% CI	u-95% CI
Depression	Family and financial	−6.18	2.5	−10.95	−1.03
Depression	Family and financial *ACE	0.73	0.26	0.2	1.24
Depression	Family and financial *ACE * Time	−0.99	0.45	−1.87	−0.1
Hostility	Intercept	10.79	5.17	0.69	20.9
Hostility	Covid Lifestyle * ACE	−0.56	0.25	−1.05	−0.06
Hostility	Substance use * ACE	0.81	0.4	0.02	1.59
Hostility	Covid Lifestyle * ACE * Time	0.14	0.06	0.02	0.27
Anxiety	Age	0.53	0.27	0.02	1.06

All comparisons in the early to peak phase and peak to post-peak phase use the pre-COVID-19 baseline to early phase as the reference category.

For hostility, the regression shows that COVID lifestyle changes and substance use both interact with ACEs to influence hostility levels. The interaction between lifestyle stress and ACEs (β = −0.56) suggests a buffering effect, where individuals with higher ACE exposure report lower hostility in response to lifestyle changes compared to those with lower ACEs. In contrast, the interaction with substance use (β = 0.81) indicates that ACEs amplify the effect of substance use on hostility, such that individuals with more ACEs experience heightened hostility when also facing increased substance use stress.

Although the two-way interaction between lifestyle stress and ACEs is negative (β = −0.56), the positive significant three-way interaction with time (β = 0.14) suggests that this relationship shifts as the pandemic progresses. Specifically, the protective (buffering) effect of ACEs in the context of lifestyle stress weakens over time, and the combined influence of ACEs and lifestyle disruptions becomes more strongly associated with hostility in later phases of the pandemic.

Moderate correlations between hostility and other mental health outcomes, such as anxiety and depression, point to shared underlying risk factors. However, the weaker correlation between hostility and perceived stress suggests that hostility may be more specifically influenced by distinct stressors, like substance use or lifestyle disruptions, rather than general perceived stress. This emphasizes that hostility may be more reactive to specific triggers rather than broad emotional or psychological distress.

Regarding anxiety, the regression highlights a positive relationship with age (β = 0.53), indicating that anxiety levels increase as individuals get older. The correlation analysis shows moderate to strong associations between anxiety, depression, and hostility, suggesting overlapping risk factors across these mental health outcomes. This is especially important given the interconnected pathways between these variables, with age emerging as a more general risk factor that affects multiple aspects of mental health during the pandemic.

## 4 Discussion

This study highlights the compounding effects of adverse childhood experiences and COVID-19 stressors on mental health outcomes in young adults, demonstrating a complex interaction between these early life stressors and pandemic-related challenges. Our longitudinal data, collected across multiple phases of the COVID-19 pandemic, provides a unique perspective on how mental health trajectories, particularly in the domains of depression, anxiety, hostility, and perceived stress, are shaped by the interaction between ACEs and COVID-19 stressors.

One of the key findings from this study is the persistent impact of high ACEs on mental health across the Early, Peak, and Post-Peak phases of the pandemic. Participants with high ACEs reported significantly worse mental health outcomes compared to those with low ACEs, particularly in terms of depression and hostility. This aligns with previous studies showing that individuals with ACEs are more vulnerable to stressors in adulthood, including the effects of the COVID-19 pandemic (19, 20). Clemens et al. (19) found that ACEs were associated with higher depressive symptoms during the pandemic, while Haydon and Salvatore (20) noted increased susceptibility to stress, particularly in younger populations. Our findings extend these insights by showing that mental health disparities in young adults with high ACEs persist throughout the different phases of the pandemic. Our findings support the continued need to screen for ACE exposure and intervene early, particularly in youth populations. Trauma-informed interventions such as trauma-focused cognitive-behavioral therapy (TF-CBT) or mindfulness-based stress reduction (MBSR) may help regulate emotional responses to stress. Routine ACE screening in primary care or University counseling centers could facilitate timely referrals to such services. The role of specific COVID-19 stressors also emerged as an important factor in understanding mental health changes. Family and financial stress was consistently associated with worse mental health outcomes, particularly for individuals with higher ACE scores. This is consistent with findings by Russo et al. (21), who reported that COVID-19-related stress, particularly financial strain, exacerbated mental health problems in individuals with high ACEs. Our study reinforces this by showing that individuals with high ACEs experience a diminished protective effect from resilience in the face of financial stress, leading to prolonged mental health challenges. Policy-level interventions, such as guaranteed income supports, tuition relief programs, or rent subsidies targeted toward vulnerable youth, could help buffer the effects of economic stress. Community organizations and public health units could also provide targeted case management or financial navigation support to reduce stress related to employment and housing insecurity.

Hostility was another mental health domain that exhibited a strong association with both ACEs and COVID-19 stressors. Our findings indicated that lifestyle changes and substance use during the pandemic significantly interacted with ACEs to elevate hostility levels, particularly during the Peak phase of the pandemic. This finding aligns with research by Killgore et al. (41), which highlighted increased aggression during COVID-19 lockdowns, especially among individuals experiencing high levels of stress. Similarly, Al-Sejari and Al-Ma'seb (42) found increased hostility and violence during the lockdowns, particularly among those facing heightened stressors. Our results extend this by identifying ACEs as a critical moderator, showing that individuals with a history of adversity are more susceptible to hostility in response to stress. This emphasizes the need for behavioral health programs that address emotion regulation and impulse control in at-risk populations. Interventions such as dialectical behavior therapy (DBT) groups, anger management programs, or substance use prevention initiatives could be delivered through community mental health agencies or virtual platforms accessible to youth.

Another key observation is the dynamic relationship between anxiety, depression, and hostility. Correlation analyses revealed that anxiety, in particular, became more tightly linked to both depression and hostility as the pandemic progressed. This pattern is consistent with findings by Stinson et al. (18) and Guo et al. (17), who reported that the psychological toll of the pandemic disproportionately affected individuals with pre-existing vulnerabilities, including those with higher ACEs. These findings are consistent with recent evidence showing elevated anxiety and depression among young adults during the pandemic, particularly in females and students (43, 44). We found that perceived stress remained stable across phases, but its strong correlation with depression suggests it still plays a central role in emotional distress during crises. The increasing co-variation of anxiety with other outcomes indicates that anxiety became a hub symptom, and one that may offer early intervention points. Public health messaging and school-based interventions should incorporate psychoeducation about the overlap of anxiety, depression, and irritability, encouraging help-seeking behaviors. Digital interventions—such as app-based CBT tools or guided relaxation platforms—can also increase access to early support, particularly for students and youth in remote or underserved areas. The multivariate mixed-effects model further illustrated that changes in mental health outcomes were not uniform but varied depending on the type of COVID-19 stressor and the level of ACEs. For instance, individuals with high ACEs showed greater mental health deterioration in response to emotional stressors, particularly during the Peak and Post-Peak phases of the pandemic. This finding is in line with Alrahdi et al. (23), who found that young adults with high ACEs and high COVID-19 stress experienced more severe mental health problems early in the pandemic. Our study extends this evidence by showing that this vulnerability persists across multiple pandemic phases, with different stressors contributing to different outcomes. These findings highlight the importance of sustained funding for post-pandemic recovery initiatives, including long-term mental health support for youth. Peer support groups, virtual mental health programs, and school-based outreach efforts should continue into the recovery period to address ongoing emotional stress, especially in those with trauma histories.

### 4.1 Strengths and limitations

A major strength of this study is its longitudinal design, which allowed us to capture the evolving mental health trajectories over several phases of the pandemic. By using baseline data from before the pandemic, we were able to establish a clearer picture of how mental health changed over time in response to both ACEs and pandemic-related stressors. Additionally, the use of a multivariate mixed-model framework allowed for a nuanced understanding of how different types of stressors interact with ACEs to impact mental health outcomes.

However, this study also has limitations. The sample size, while sufficient for detecting moderate effects, may limit the generalizability of our findings to broader populations. Additionally, while we captured a range of COVID-19 stressors, there may be other unmeasured factors, such as social support or pre-existing mental health conditions, that could further explain the observed mental health trajectories. Finally, the reliance on self-reported data for both ACEs and mental health outcomes introduces the possibility of response bias.

## 5 Conclusion

This study establishes a clear link between elevated COVID-19 stressors and heightened mental health issues among young adults in Canada who report significant childhood adversity. Prior research has consistently demonstrated associations between high ACEs and COVID-19-related stressors on mental health during the early phase of the pandemic among adolescents (17, 18, 20), adults (19–21), and young adults (23). Our findings not only corroborate these earlier studies but also extend them by showing that the negative impact on mental health persisted—and even intensified—across the Early, Peak, and Post-Peak phases of the pandemic. This suggests that individuals with high ACEs may face long-term mental health vulnerabilities, particularly during prolonged periods of stress like the COVID-19 pandemic.

Young adults are in a unique life stage where they may be disproportionately affected by government containment measures, economic disruptions, and social isolation caused by the pandemic (45). This life stage vulnerability, combined with childhood adversity, may have lasting effects on their mental health as they age. Time will reveal whether the mental health challenges faced by this cohort during the pandemic will have a long-lasting legacy or whether these effects will eventually subside. However, based on previous research, individuals with higher ACEs have been shown to experience more significant mental and physical health issues later in life (6). Whether the current cohort of young adults with high ACEs will experience similar or even more pronounced long-term health problems remains an open question that warrants further longitudinal investigation.

Additionally, the study highlights that young adults with a history of childhood exposure to abuse and severe household dysfunction are more vulnerable to subsequent stressors as they transition into adulthood. Previous studies have suggested that individuals with high ACEs may face a greater risk of exposure to adult stressors, such as job loss or relationship instability (2), or that they may be more vulnerable to these stressors when they do occur. This increased vulnerability could be due to reduced stability in adult roles and a diminished capacity to cope with later-life stressors (26). Alternatively, the biological embedding of stress, as suggested by Miller et al. (46) and De Bellis and Zisk (47), may lead to a heightened physiological stress response, contributing to both mental and physical health problems later in life.

This high-ACEs group appears to be doubly disadvantaged: they may not only experience more stressors over the course of their lives but also react more intensely to those stressors, which could result in a greater cumulative burden of health problems. The findings of this research underscore the importance of identifying young adults with high ACEs as a critical subgroup that may benefit from targeted interventions aimed at mitigating the adverse mental health outcomes associated with both childhood adversity and ongoing stress. Furthermore, addressing their mental health challenges early on may also help prevent future physical health problems, as prolonged stress reactivity is known to contribute to a range of chronic conditions.

## Data Availability

The raw data supporting the conclusions of this article will be made available by the authors, without undue reservation.
