# Supplementary material for: Mental health trajectories over the COVID-19 pandemic among young adults reporting adverse childhood experiences
Source: Front Public Health. 2025 Jul 9;13:1546409. doi: 10.3389/fpubh.2025.1546409 (PMC12283320; doi:10.3389/fpubh.2025.1546409)
Supplement: Supplementary file 1 [file Data_Sheet_1.PDF]

Supporting Information

Table S1: Factor loadings of COVID-19 stressors based on an EFA of the Early Phase of COVID-19 Data

COVID-19 Stressor Scale Items	COVID-19 Stress Factor Loadings				
	Emotional	Family & financial	Lifestyle	Community support	Substance use
I have felt nervous, anxious, or worried	**.783**			.213	
I have felt sad, depressed, or hopeless	**.810**	.126	.201		
I have felt lonely and isolated	**.700**	.313	.283		.106
I have felt irritable, frustrated, or angry	**.726**	.158	.256	.145	
I have felt suspicious and distrustful of others	**.630**		-.245	.180	.142
I have had my job hours cut/lost income	.199	**.604**	.130		
I have been unable to pay my rent/mortgage		**.827**		.190	.146
I have been unable to pay for food		**.806**		.323	.142
I have had serious arguments with the people I live with	.354	**.484**	.094	.199	-.251
I have had serious physical fights with the people I live with	.116	**.507**	-.171	-.169	-.394
I have been less physically active	.130		**.884**	.157	
My diet has gotten worse	.205		**.843**		.104
I have not been able to get food and supplies that I need				**.713**	
People in my community are acting inconsiderately/selfishly	.312	.146	.143	**.574**	.109
I have had trouble getting non-COVID-19 related medical	.307	.114	.104	**.698**	-.102
I have increased how often/how much I use recreational drugs (e.g., marijuana, cocaine, opioids, etc.)				.100	**.761**
I have increased how often/how much I drink alcohol	.206	.212	.174	-.225	**.659**

Table S2: Attrition analysis for the Mental Health, ACEs, and covariate measures across all COVID-19 Sub-study phases (Baseline N= 248)

	Early		Peak		Post-Peak		P-Value
	responders	non-responders	responders	non-responders	responders	non-responders	
**Mental health**							
Depression	171	77	161	87	138	110	0.35
Anxiety	171	77	161	87	138	110	0.26
Hostility	171	77	161	87	138	110	0.13
Perceived Stress	171	77	161	87	138	110	0.28
**ACEs**	171	77					0.10
**Covariates**							
Sex	171	77					0.08
Age	171	77					0.81
Education	171	77					0.01

Table S3: Results of the Bayesian multivariate mixed effect regression model on the mental health changes across the pandemic phases.

Mental health	Covariates	Beta	SE	L-95% CI	U-95% CI
Depression	Intercept	36.26	20.16	-3.09	76.14
	Male	0.39	0.98	-1.52	2.37
	Education	-0.06	0.44	-0.91	0.77
	Age	0.36	0.52	-0.66	1.37
	Emotional	0.73	1.91	-3	4.55
	Family & Financial	-6.18	2.5	-10.95	-1.03
	Lifestyle	-1.41	2.43	-6.2	3.43
	Community support	2.09	3.62	-5.07	9.16
	Substance use	4.91	4.83	-4.86	14.34
	ACES	-5.12	2.7	-10.44	0.16
	Time	-4.75	4.52	-13.37	4.13
	Emotional * Time	-0.19	0.48	-1.13	0.75
	Family & Financial * Time	1.26	0.65	-0.11	2.53
	Lifestyle * Time	0.26	0.61	-0.97	1.45
	Community support * Time	-0.51	0.92	-2.3	1.32
	Substance use * Time	-1.48	1.25	-3.91	1.06
	Emotional * ACES	-1.04	0.79	-2.57	0.54
	Family & Financial * ACES	0.73	0.26	0.2	1.24
	Lifestyle * ACES	0.61	1	-1.33	2.57
	Community support * ACES	-1.14	1.42	-3.93	1.66
	Substance use * ACES	-1.59	1.62	-4.73	1.62
	Emotional * ACES * Time	0.21	0.2	-0.18	0.59
	Family & Financial * ACES * Time4	-0.38	0.23	-0.82	0.07
	Family & Financial * ACES * Time5	-0.99	0.45	-1.87	-0.1
	Lifestyle * ACES * Time	-0.12	0.26	-0.62	0.38
	Community support * ACES * Time	0.29	0.37	-0.44	1
	Substance use * ACES * Time	0.52	0.42	-0.31	1.33
Hostility	Intercept	10.79	5.17	0.69	20.9
	Male	0.1	0.25	-0.39	0.58

	Education	-0.01	0.11	-0.23	0.21
	Age	0.09	0.13	-0.18	0.34
	Emotional	0.12	0.47	-0.81	1.01
	Family & Financial	-0.29	0.62	-1.51	0.92
	Lifestyle	0.14	0.61	-1.04	1.33
	Community support	0.06	0.93	-1.73	1.85
	Substance use	-1.84	1.17	-4.14	0.46
	ACES	-0.27	0.71	-1.71	1.13
	Time	-2	1.14	-4.21	0.29
	Emotional * Time	-0.02	0.12	-0.25	0.2
	Family & Financial * Time	0.02	0.16	-0.3	0.34
	Lifestyle * Time	-0.04	0.15	-0.34	0.26
	Community support * Time	-0.01	0.24	-0.46	0.45
	Substance use * Time	0.39	0.31	-0.2	1
	Emotional * ACES	-0.25	0.2	-0.64	0.13
	Family & Financial * ACES	0.03	0.07	-0.1	0.16
	Lifestyle * ACES	-0.56	0.25	-1.05	-0.06
	Community support * ACES	-0.09	0.36	-0.78	0.63
	Substance use * ACES	0.81	0.4	0.02	1.59
	Emotional * ACES * Time	0.05	0.05	-0.04	0.15
	Family & Financial * ACES * Time4	-0.05	0.06	-0.17	0.07
	Family & Financial * ACES * Time5	-0.1	0.12	-0.32	0.13
	Lifestyle * ACES * Time	0.14	0.06	0.02	0.27
	Community support * ACES * Time	0.03	0.09	-0.16	0.21
	Substance use * ACES * Time	-0.17	0.1	-0.38	0.03
Anxiety	Intercept	0.86	10.63	-19.87	21.22
	Male	-0.11	0.5	-1.09	0.88
	Education	-0.19	0.22	-0.62	0.25
	Age	0.53	0.27	0.02	1.06
	Emotional	-0.32	0.97	-2.28	1.56
	Family & Financial	-1.06	1.29	-3.63	1.45
	Lifestyle	-0.97	1.24	-3.35	1.45
	Community support	0.95	1.89	-2.76	4.68

	Substance use	1.76	2.43	-3.02	6.44
	ACES	-2.32	1.4	-5.08	0.3
	Time	0.04	2.39	-4.61	4.78
	Emotional * Time	0.14	0.24	-0.33	0.63
	Family & Financial * Time	0.21	0.34	-0.44	0.88
	Lifestyle * Time	0.16	0.31	-0.44	0.77
	Community support * Time	-0.15	0.48	-1.1	0.78
	Substance use * Time	-0.79	0.63	-2.01	0.46
	Emotional * ACES	-0.63	0.41	-1.41	0.18
	Family & Financial * ACES	0.23	0.14	-0.04	0.5
	Lifestyle * ACES	-0.1	0.51	-1.11	0.85
	Community support * ACES	0.25	0.74	-1.23	1.72
	Substance use * ACES	-0.03	0.83	-1.65	1.57
	Emotional * ACES * Time	0.11	0.1	-0.1	0.31
	Family & Financial * ACES * Time4	-0.19	0.12	-0.43	0.05
	Family & Financial * ACES * Time5	-0.35	0.24	-0.83	0.1
	Lifestyle * ACES * Time	0.07	0.13	-0.19	0.33
	Community support * ACES * Time	-0.08	0.19	-0.45	0.3
	Substance use * ACES * Time	0.17	0.21	-0.25	0.58
Perceived Stress	Intercept	16.86	11.07	-4.81	38.53
	Male	0.41	0.52	-0.64	1.45
	Education	-0.08	0.24	-0.54	0.38
	Age	0.03	0.28	-0.51	0.58
	Emotional	-0.13	1	-2.1	1.72
	Family & Financial	-2.12	1.36	-4.75	0.5
	Lifestyle	-0.39	1.35	-2.99	2.21
	Community support	0.71	1.99	-3.15	4.54
	Substance use	1.35	2.55	-3.73	6.31
	ACES	-2.67	1.45	-5.5	0.19
	Time	-1.48	2.46	-6.13	3.38
	Emotional * Time	0.06	0.25	-0.41	0.56
	Family & Financial * Time	0.4	0.35	-0.28	1.09
	Lifestyle * Time	0.09	0.34	-0.56	0.74

	Community support * Time	-0.28	0.51	-1.25	0.71
	Substance use * Time	-0.49	0.66	-1.77	0.86
	Emotional * ACES	-0.6	0.42	-1.42	0.22
	Family & Financial * ACES	0.23	0.14	-0.05	0.51
	Lifestyle * ACES	0.31	0.55	-0.77	1.35
	Community support * ACES	-0.09	0.77	-1.58	1.43
	Substance use * ACES	0.07	0.88	-1.64	1.83
	Emotional * ACES * Time	0.09	0.11	-0.12	0.3
	Family & Financial * ACES * Time4	-0.13	0.13	-0.37	0.12
	Family & Financial * ACES * Time5	-0.26	0.25	-0.73	0.23
	Lifestyle * ACES * Time	-0.08	0.14	-0.34	0.2
	Community support * ACES * Time	0.11	0.2	-0.27	0.5
	Substance use * ACES * Time	0.04	0.23	-0.4	0.47

All significant results are highlighted.
